# Correction: Fisher, Aron B., et al. A Peptide Inhibitor of NADPH Oxidase (NOX2) Activation Markedly Decreases Mouse Lung Injury and Mortality Following Administration of Lipopolysaccharide (LPS). *Int. J. Mol. Sci.* 2019, *20*, 2395

**DOI:** 10.3390/ijms21020398

**Published:** 2020-01-08

**Authors:** Aron B. Fisher, Chandra Dodia, Shampa Chatterjee, Sheldon I. Feinstein

**Affiliations:** Institute for Environmental Medicine and Department of Physiology, University of Pennsylvania Perelman School of Medicine, Philadelphia, PA 19104, USA; cdodia@pennmedicine.upenn.edu (C.D.); shampac@pennmedicine.upenn.edu (S.C.); sif@pennmedicine.upenn.edu (S.I.F.)

The authors wish to make the following corrections to our previously published paper [1]. 

Because of partially incorrect data plotted by error, replace old Figure 2

with new Figure 2.

Because of publication of the incorrect figure, replace old Figure 5

with new Figure 5.

## Figures and Tables

**Figure 2:**
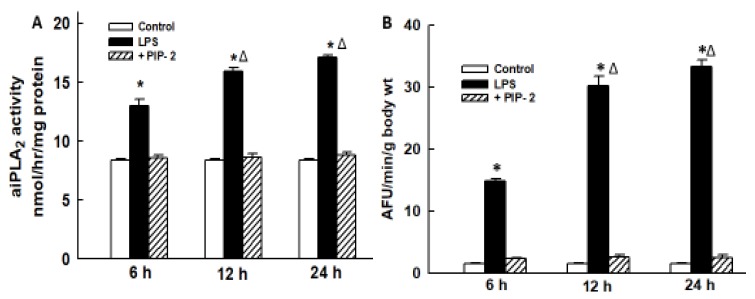
PIP-2 inhibits the increased lung aiPLA2 activity and increased ROS generation after LPS administration. LPS (5 µg/g body weight) was administered by intratracheal (IT) instillation along with liposomes alone (labeled as LPS) or with PIP-2 in liposomes (labeled as +PIP-2). Control was liposomes alone without LPS (labeled as control). Mice were sacrificed at 6, 12, or 24 h after LPS and lungs were perfused in situ for 15 min with saline solution containing the fluorophore difluorofluoroscein diacetate (DFF-DA). Lungs were then homogenized and assayed for (**A**) aiPLA_2_ activity; and (**B**) fluorescence of the lung homogenate as an index of ROS production. Results are mean ± SE for *n* = 3 for (**A**) and *n* = 4 for (**B**). * *p* < 0.05 vs. corresponding control and corresponding +PIP-2 values at the same time point; ^Δ^ *p* < 0.05 vs. the corresponding value at 6 h.

**Figure 2:**
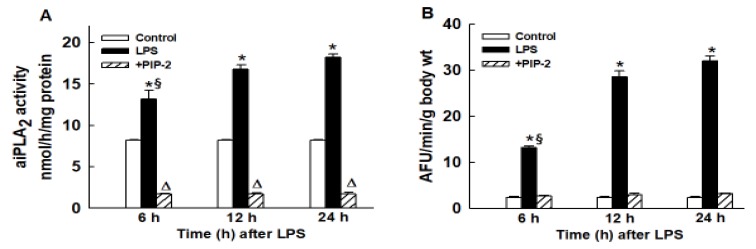
PIP-2 inhibits the increased lung aiPLA2 activity and increased ROS generation that follows LPS administration. LPS (5 µg/g body weight) was administered by intratracheal (IT) instillation along with liposomes alone (labeled as LPS) or with PIP-2 in liposomes (labeled as +PIP-2). Control was liposomes alone without LPS (labeled as control). Mice were sacrificed at 6, 12, or 24 h after LPS and some lungs were perfused in situ for 15 min with saline solution (**A**) while others were perfused in situ for 15 min with saline solution containing the fluorophore difluorofluoroscein diacetate (DFF-DA) (**B**). Lungs were then homogenized and assayed for aiPLA_2_ activity (**A**), or fluorescence of the lung homogenate as an index of ROS production (**B**). Results are mean ± SE for *n* = 3 for (**A**) and *n* = 4 for (**B**). * *p* < 0.05 vs. both the corresponding control and the corresponding LPS+PIP-2 values at the same time point; § *p* < 0.05 vs. 12 h and 24 h LPS values; Δ *p* < 0.05 vs. corresponding control value.

**Figure 5:**
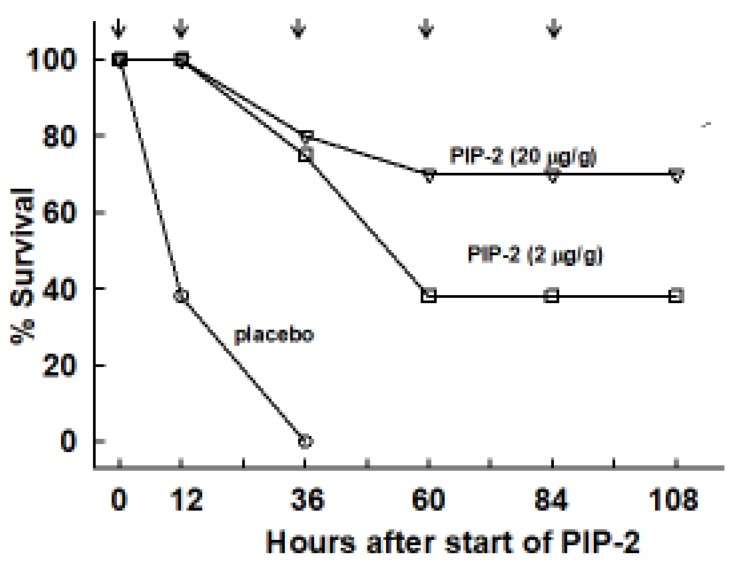
PIP-2 prevents mouse mortality with high dose LPS. Mice were administered LPS (15 µg/g body weight) by intratracheal instillation and divided into two groups. At 12 h after LPS, one group was given PIP-2 in liposomes by intravenous injection (IV) while the other group (placebo) was given liposomes alone. The time of the treatment initiation (12 h after LPS) is plotted as zero time. Treatment was repeated at 12, 36, 60, and 84 h after the initial dose of PIP-2,co-incident with the plotted points. Surviving mice were sacrificed at 108 h. *n* = 12 for placebo and *n* = 11 for PIP-2.

**Figure 5:**
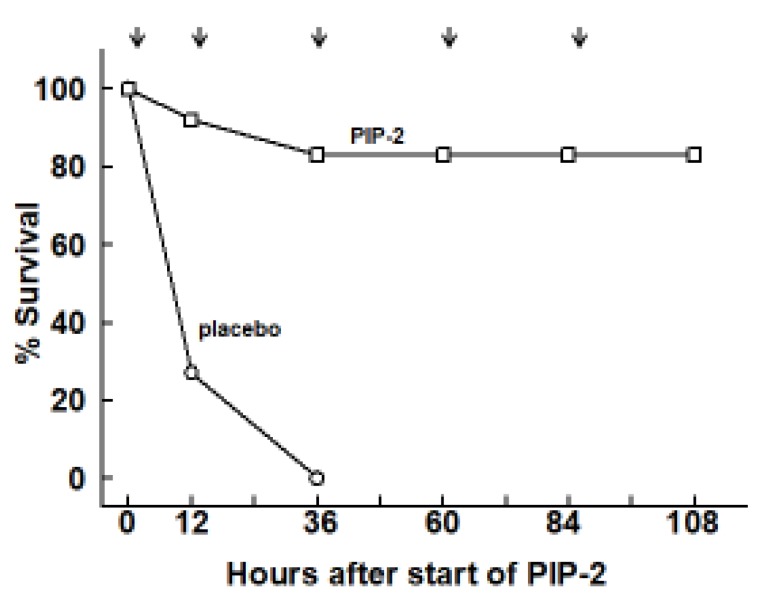
PIP-2 prevents mouse mortality with high dose LPS. Mice were administered LPS (15 µg/g body weight) by intratracheal instillation and divided into two groups. At 12 h after LPS, one group was given PIP-2 (2 µg/g body weight) in liposomes by intravenous injection (IV) while the other group (placebo) was given liposomes alone. The time of treatment initiation (12 h after LPS) is plotted as zero time. Treatment was repeated at 12, 36, 60, and 84 h after the initial dose of PIP-2, as indicated by the arrows. Surviving mice were sacrificed at 108 h after start of PIP-2 (120 h after LPS). *n* = 12 for placebo and *n* = 11 for PIP-2.
